# Circulating nephrin autoantibodies and posttransplant recurrence of primary focal segmental glomerulosclerosis

**DOI:** 10.1111/ajt.17077

**Published:** 2022-05-09

**Authors:** Motoshi Hattori, Yoko Shirai, Shoichiro Kanda, Kiyonobu Ishizuka, Naoto Kaneko, Taro Ando, Makoto Eguchi, Kenichiro Miura

**Affiliations:** ^1^ Department of Pediatric Nephrology Tokyo Women’s Medical University Tokyo Japan; ^2^ Department of Pediatrics The University of Tokyo Tokyo Japan

## FUNDING INFORMATION

This work was supported in part by Grants‐in‐Aid Scientific Research (C) (JP18K07857 [MH], JP21K07829 [KM], and JP21K07857 [NK]) from the Ministry of Education, Culture, Sports, Science and Technology of Japan.

## DISCLOSURE

The authors of this manuscript have no conflicts of interest to disclose as described by the *American Journal of Transplantation*.

*To the Editor:*

Posttransplant recurrence of primary focal segmental glomerulosclerosis (FSGS) is a major challenge in the field of kidney transplantation. Primary FSGS is thought to be caused by circulating factors (CFs) that injure podocytes,^1^ but these factors remain unknown despite intensive efforts to identify them.^2^ Recently, one posttransplant recurrent FSGS patient with pretransplant nephrin autoantibodies was reported; however, the involvement of nephrin autoantibodies in the pathogenesis of recurrence has not been clarified.^3^ Here, we present very early posttransplant recurrence changes of podocytes and possible roles of circulating nephrin autoantibodies in FSGS recurrence.

This study was approved by the local Ethics Committee of Tokyo Women’s Medical University (approval no. 2021–0184). We analyzed plasma samples and kidney tissue specimens of preperfusion (0 h) and postperfusion (1 h) in one patient (patient 1) with and one (patient 2) without FSGS recurrence after first kidney transplantation. Patient 1 had no pathogenic variants in FSGS‐related genes including *NPHS1*. Patient 2 was shown to carry compound heterozygous *LAMB2* variants after his kidney transplantation. Plasma samples were obtained before any prophylactic therapies were administered for their kidney transplantation. Patient clinical data are presented in Table S1 and methods are provided in the Supplementary Methods.

**FIGURE 1:**
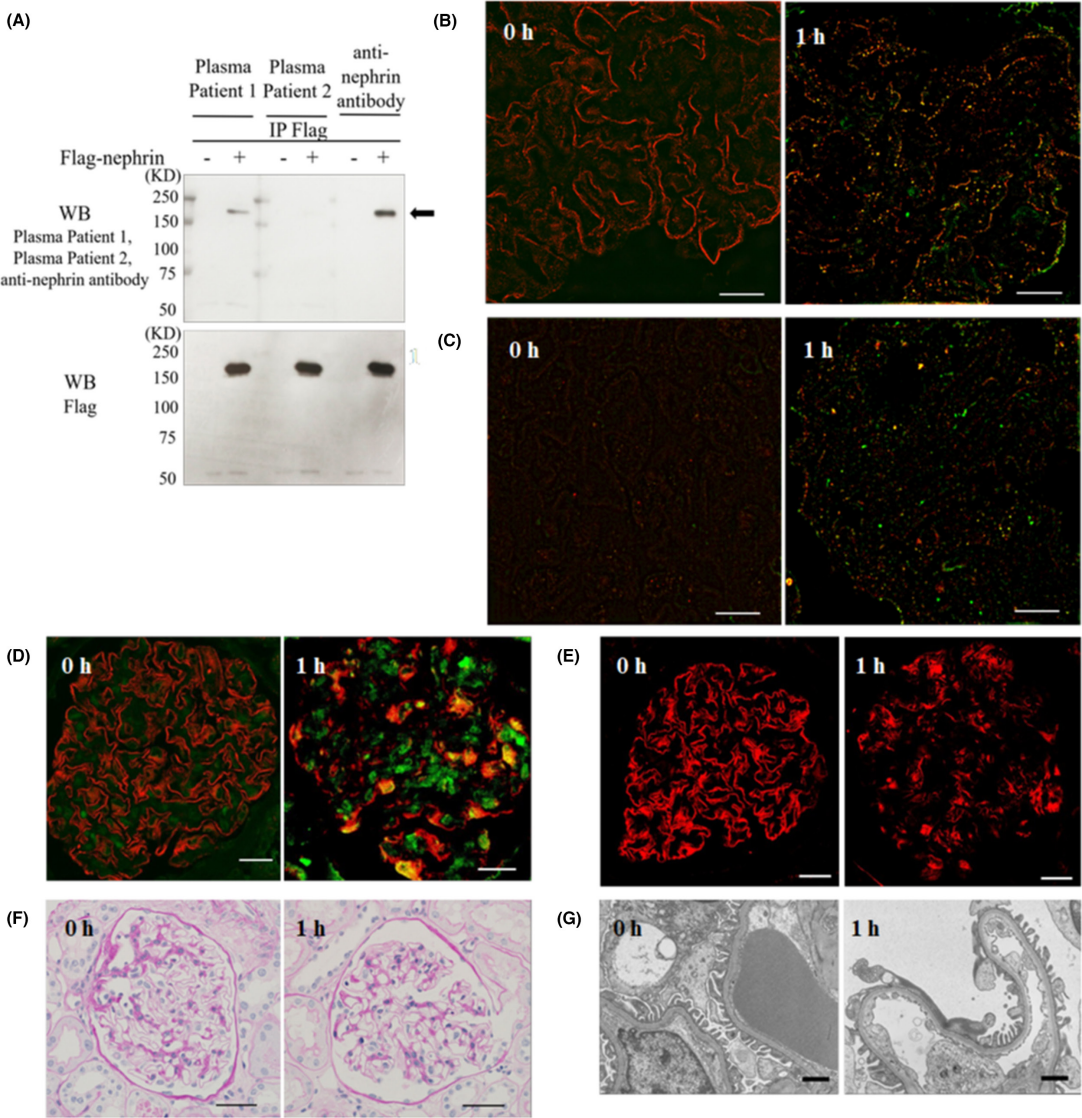
Presence of autoantibodies to nephrin and early podocyte changes in the posttransplant recurrence of focal segmental glomerulosclerosis (FSGS). Western blot analysis of nephrin using plasma from patients with and without FSGS recurrence and representative immunopathological images of preperfusion (0 h) and postperfusion (1 h) biopsy specimens from the FSGS recurrent patient. (A) Western blot analysis of Flag‐nephrin using patients’ plasma. Plasma from the recurrent patient (patient 1) recognized Flag‐nephrin protein (arrow). (B) Structured illumination microscopy (SIM) from the recurrent patient. A clear overlap (yellow) of punctate IgG (green) with nephrin (red) is observed in the 1 h biopsy specimen. Scale bar, 10 μm. Additional images are provided in Figure S1. (C) SIM images after dual staining for IgG (green) and phosphorylated (p)‐nephrin^Y1176^ (red) from the recurrent patient showing induced nephrin tyrosine phosphorylation colocalizing with IgG (yellow) in the 1 h biopsy specimen. Scale bar, 10 μm. Additional images are provided in Figure S2. (D) Confocal microscopy images after dual staining for nephrin (red) and ShcA (green) from the recurrent patient showing upregulated ShcA expression colocalizing with nephrin (yellow) in the 1 h biopsy specimen. Scale bar, 20 μm. Additional images are provided in Figure S3. (E) Immunofluorescence of nephrin expression in the recurrent patient showing a change from linear staining (0 h biopsy) to granular staining (1 h biopsy); scale bar, 20 μm. (F) Periodic acid–Schiff‐stained light micrographs showing normal glomeruli in the 0 h and 1 h biopsies from the recurrent patient; scale bar, 50 μm. (G) Electron micrographs showing podocyte foot process effacement in the 1 h biopsy from the recurrent patient; scale bar, 2 μm. Additional data are provided in Figure S4.

We identified autoantibodies to nephrin in plasma of the patient showing recurrence (patient 1), but not in plasma of the patient without recurrence (patient 2) (Figure 1A). We further observed that punctate IgG was present and specifically colocalized with nephrin in the 1 h biopsy from the FSGS recurrent patient (Figure 1B and Figure S1), which is in line with the findings of Watts et al. for a subset of minimal change disease biopsies.^3^

In a previous in vitro experiment, a monoclonal anti‐nephrin antibody induced nephrin tyrosine phosphorylation.^4^ Moreover, Src homology and collagen homology A (ShcA) protein was found to be expressed at low levels in podocytes and to bind tyrosine‐phosphorylated nephrin as an adaptor protein.^5^ Here, dual immunofluorescence (IF) staining for IgG and phosphorylated (p)‐nephrin^Y1176^ revealed induced nephrin tyrosine phosphorylation colocalizing with IgG in the 1 h biopsy from the recurrent patient (Figure 1C and Figure S2). We further observed increased expression of ShcA in the 1 h biopsy from the recurrent patient and observed it to colocalize with nephrin (Figure 1D and Figure S3). These findings indicate that nephrin tyrosine phosphorylation was induced very early after exposure of circulating nephrin autoantibodies.

Nephrin tyrosine phosphorylation is known to influence signal transduction pathways which control several podocyte functions including nephrin trafficking and cytoskeletal organization.^5^ In this study, IF staining for nephrin showed linear expression along the glomerular capillary in the 0 h biopsy from the recurrent patient, but nephrin expression took on a granular pattern in the 1 h biopsy (Figure 1E). Moreover, glomeruli appeared normal under light microscopy in the 1 h biopsy (Figure 1F), but podocyte foot process effacement (FPE) was segmentally observed under electron microscopy in the 1 h biopsy from the recurrent patient (Figure 1G and Figure S4).

Further studies are clearly needed to confirm our preliminary results; however, our current findings indicate that circulating nephrin autoantibodies detected in plasma from a recurrent patient induce nephrin tyrosine phosphorylation that leads to increased ShcA expression, altered nephrin distribution, and podocyte FPE very early after kidney transplantation. Furthermore, circulating nephrin autoantibodies could be a possible candidate for CFs in the pathogenesis of recurrent FSGS.

## Supporting information

Supplementary Material
