# Supplementary material for: Circulating nephrin autoantibodies and posttransplant recurrence of primary focal segmental glomerulosclerosis
Source: Am J Transplant. 2022 May 9;22(10):2478–80. doi: 10.1111/ajt.17077 (PMC9790549; doi:10.1111/ajt.17077)
Supplement: Supplementary file 1 — Supplementary Material [file AJT-22-2478-s001.doc]

**Supplemental Material**

Supplement to: Hattori M, Shirai Y, Kanda S, Ishizuka K, Kaneko N, Ando T, Eguchi M and Miura K. Circulating nephrin autoantibodies and post-transplant recurrence of primary focal segmental glomerulosclerosis.

**Table S1.** Patient demographic and clinical data. p2

**Supplementary methods.** p4

**Additional references.** p7

**Supplementary Figure Legends** p9

**Figure S1.** Structured illumination microscopy images after dual staining for nephrin and IgG from patients with and without recurrence. P11

**Figure S2.** Structured illumination microscopy images after dual staining for IgG and phospholylated-nephrin^Y1176^ from patients with and without recurrence. P12

**Figure S3.** Confocal images after dual staining for nephrin and ShcA from patients with and without recurrence. P13

**Figure S4.** Foot process width of patients with and without recurrence. P14

**Table S1.** **Patient demographic and clinical data**

|  | **Recurrence*** | |  | **Non-recurrence** |
| --- | --- | --- | --- | --- |
|  |  | **Patient 1**† |  | **Patient 2**† |
| Sex |  | F |  | M |
| Race |  | Japanese |  | Japanese |
| Age at onset of disease (years) |  | 1.8 |  | 1.8 |
| Family history |  | None |  | None |
| Nephrotic syndrome |  | Yes |  | Yes |
| Serum albumin at onset (g/dL) |  | 0.9 |  | 1.5 |
| Urine protein excretion at onset (g/gCr) |  | 22.0 |  | 28.5 |
| Responses to steroid and immunosuppressive therapies* |  | PR |  | NR |
| Histological diagnosis |  | FSGS |  | FSGS |
| Degree of foot process effacement* |  | Diffuse |  | Segmental |
| Time from onset to ESKD (years) |  | 0.5 |  | 0.3 |
| Duration of dialysis before transplantation (years) |  | 5.0 |  | 5.2 |
| Native nephrectomy |  | No |  | No |
| Age at kidney transplantation (years) |  | 7.2 |  | 7.3 |
| Serum albumin at the time of transplantation (g/dL) |  | 4.1 |  | 3.7 |
| Urine protein excretion at the time of transplantation (g/gCr) |  | 0‡ |  | 0‡ |
| Preemptive transplantation |  | No |  | No |
| Second transplantation |  | No |  | No |
| Donor type |  | LD |  | LD |
| Prophylactic plasmapheresis |  | Yes (4 sessions) |  | Yes (3 sessions) |
| Induction therapy for kidney transplantation |  | Basiliximab |  | Basiliximab |
| Immunosuppressive regimen |  | Tac, MMF, mPSL |  | Tac, MMF, mPSL |
| Time to recurrence (days) |  | 1 |  | Not applicable |
| Maximum proteinuria at the time of recurrence (g/gCr) |  | 31.5 |  | Not applicable |

F, female; M, male; PR, partial remission; NR, no remission; FSGS, focal segmental glomerulosclerosis; ESKD, end-stage kidney disease; LD, living donor; Tac, tacrolimus; MMF, mycophenolate mofetil; mPSL, methylprednisolone; Cr, creatinine

*Definitions of post-transplant recurrence of FSGS, the responses to steroid and immunosuppressive therapies, and the degree of foot process effacement have been previously described^1-4^. Briefly, post-transplant recurrence was defined as: (i) the occurrence of nephrotic-range proteinuria (urine protein creatinine ratio [UPCR] >2 g/g) after kidney transplantation, and (ii) diffuse foot process effacement (FPE) in graft biopsy on electron microscopy (EM) ^1, 2^. Complete remission, partial remission (PR), and no remission (NR) were defined as UPCR <0.2 g/g or negative or trace dipstick on three consecutive occasions, UPCR between 0.2 g/g and 2.0 g/g, and UPCR >2.0 g/g or a lack of reduction in urine protein excretion by 40%, respectively^2, 3^. Diffuse FPE on EM was defined as >80% FPE^4^.

†Patient 1 had no pathogenic variants in FSGS-related genes including *NPHS1*^2^. Patient 2 was shown to carry compound heterozygous *LAMB2* variants after his kidney transplantation^5^**.**

‡Both patients were anuric at the time of transplantation.

**Supplementary Methods**

This study was conducted in accordance with the Declaration of Helsinki. All participants or their legal guardians gave their written informed consent. The clinical and research activities reported here are consistent with the Principles of the Declaration of Istanbul as outlined in the “Declaration of Istanbul on Organ Trafficking and Transplant Tourism”.

**Kidney biopsy and pathology evaluation**

Wedge kidney transplant biopsies were obtained in the operating room at preperfusion and at 1 h postperfusion of the grafted kidney just before wound closure, which is in line with the previous report ^6^.

Kidney biopsy specimens for light microscopy were fixed in 10% buffered formalin, embedded in paraffin, and stained with hematoxylin and eosin, Masson trichrome, periodic acid–Schiff, and periodic acid–silver methenamine^1^. For electron microscopy, kidney biopsy specimens were fixed in 2.5% glutaraldehyde and 1% osmium tetroxide, and embedded in Epon 812^1^.

**Immunofluorescence studies**

Immunofluorescence (IF) studies were performed as previously reported with some modifications^7^. Briefly, 3-μm-thick sections were deparaffinized in xylene and rehydrated through an ethanol-H_2_ gradient. Autoclave antigen retrieves were performed in Tris-ethylenediaminetetraacetic acid (EDTA) buffer, pH 7.8 at 125°C for 15 min. An anti-nephrin antibody (Anti-Human Nephrin (C) Rabbit IgG Affinity Purify, Immuno-Biological Laboratories, Fujioka, Japan) was used at a dilution of 1:50.

Dual IF staining for nephrin and IgG and for IgG and phosphorylated-nephrin^Y1176^ were performed using an anti-nephrin antibody (Anti-Human Nephrin (C) Rabbit IgG Affinity Purify, Immuno-Biological Laboratories) at a dilution of 1:50, goat anti-human IgG (H+L) cross-adsorbed secondary antibody, Alexa Fluor 488 (Thermo Fisher Scientific, Waltham, MA) at a dilution of 1:50, and a rabbit polyclonal anti-phospho-Nephrin (Tyr1176) antibody (PA5-105709, Thermo Fisher Scientific) at a dilution of 1:50. Image acquisition was performed in the two-dimensional structured illumination microscopy (SIM) mode with a Nikon microscope (N-SIM; Nikon, Tokyo, Japan), and image reconstruction was carried out using NIS-Elements software (Nikon) based on a previous report^8^ with kind support from Dr. Noriko Tokai of the Imaging Core Laboratory (Institute of Medical Science, The University of Tokyo, Tokyo, Japan).

Dual IF staining for nephrin and ShcA was performed using an anti-nephrin antibody (Anti-Human Nephrin (C) Rabbit IgG Affinity Purify, Immuno-Biological Laboratories) at a dilution of 1:50 and an anti-ShcA antibody (anti-ShcA mouse monoclonal antibody, Santa Cruz Biotechnology, Dallas, TX) at a dilution of 1:100. Goat anti-rabbit IgG, superclonal recombinant secondary antibody, Alexa Fluor 568 (Thermo Fisher Scientific), and goat anti-mouse IgG, superclonal recombinant secondary antibody, Alexa Fluor 488 (Thermo Fisher Scientific) were used at dilutions of 1:1000. Images were acquired on a ZEISS LSM 710 confocal microscope (Zeiss, Oberkochen, Germany).

**Cell culture and transfection**

Human embryonic kidney HEK293T cells were purchased from the American Type Culture Collection (Manassas, VA). Cells were maintained in Dulbecco’s modified Eagle’s medium containing 10% fetal calf serum. Transfections were performed using Lipofectamine 2000 (Invitrogen, Carlsbad, CA). The expression vector for human Flag-nephrin (Nephrin [NPHS1] [NM_004646] Human Tagged ORF Clone, RC217677, OriGene Technologies, Inc., Rockville, MD) was obtained commercially.

**Immunoprecipitation and western blotting**

Cells were lysed with lysis buffer (20 mM Tris-HCl [pH 7.4], 150 mM NaCl, 1% Nonidet P-40 [NP40], 1 mM EDTA, 1 mM phenylmethylsulfonyl fluoride, 50 mM NaF, 10 μg/ml antipain, 10 μg/ml leupeptin, 10 μg/ml aprotinin, 1 mM sodium vanadate) for 15 min on ice. Lysates were clarified by centrifugation, and incubated with anti-Flag M2 agarose beads (A2220, Sigma-Aldrich, St. Louis, MO) for 1 h. Beads were washed five times with Tris-buffered saline–1% NP40, and bound proteins were eluted with 100 mM glycine–HCl (pH 2.6) and analyzed by western blotting. Western blotting was performed as previously described^9^. Immunoprecipitates were run on 7.5% polyacrylamide gels and blotted onto nitrocellulose membranes, which were then incubated with patient plasma diluted 1:5000 or anti-nephrin antibody (Nephrin (NPHS1) Mouse Monoclonal Antibody, CF813415, OriGene Technologies, Inc.) diluted 1:5000 overnight at 4°C. After washing, the membranes were incubated with anti-human IgG (Fab′)2 or anti-mouse IgG for 1 h. Protein bands were visualized on the detection system.

**Additional References**

1. Hattori M, Akioka Y, Chikamoto H, et al. Increase of integrin-linked kinase activity in cultured podocytes upon stimulation with plasma from patients with recurrent FSGS. Am J Transplant 2008;8:1550-1556.

2. Miura K, Ando T, Kanda S, et al. Response to steroid and immunosuppressive therapies may predict post-transplant recurrence of steroid-resistant nephrotic syndrome [published online ahead of print July 26, 2021]. Pediatr Transplant Doi:10.1111/petr.14103.

3. Ban H, Miura K, Kaneko N, et al. Amount and selectivity of proteinuria may predict the treatment response in post-transplant recurrence of focal segmental glomerulosclerosis: a single-center retrospective study. Pediatr Nephrol 2021;36:2433-2442.

4. Ishizuka K, Miura K, Hashimoto T, et al. Degree of foot process effacement in patients with genetic focal segmental glomerulosclerosis: a single-center analysis and review of the literature. Sci Rep 2021;11:12008.

5. Kikkawa Y, Hashimoto T, Takizawa K, et al. Lamin β2 variants associated with isolated nephropathy that impact matrix regulation. JCI insight 2021;6:e145908.

6. Chang JW, Pardo V, Sageshima J, et al. Podocyte foot process effacement in postperfusion allograft biopsies correlates with early recurrence of proteinuria in focal segmental glomerulosclerosis. Transplantation 2012;93:1238-1244.

7. Hashimoto T, Harita Y, Takizawa K, et al. *In vivo* expression of NUP93 and its alteration by *NUP93* mutations causing focal segmental glomerulosclerosis. Kidney Int Rep 2019;4:1312-1322.

8. Gustafsson MG. Surpassing the lateral resolution limit by a factor of two using structured illumination microscopy. J Microsc. 2000;198:82-87.

9. Kanda S, Harita Y, Shibagaki Y, et al. Tyrosine phosphorylation-dependent activation of TRPC6 regulated by PLC-γ1 and nephrin: effect of mutations associated with focal segmental glomerulosclerosis. Mol Biol Cell. 2011;22:1824-1835.

**Supplementary Figure Legends**

**Figure S1.** **Structured illumination microscopy images after dual staining for nephrin (red) and IgG (green) from patients with and without recurrence.** A clear overlap (yellow) of punctate IgG with nephrin is observed in the 1 h biopsy from the recurrent patient (patient 1). Punctate IgG staining is not seen in the patient without recurrence (patient 2). Scale bar, 10 μm.

**Figure S2.** **Structured illumination microscopy images after dual staining for IgG (green) and phosphorylated (p)-nephrin^Y1176^ (red) from patients with and without recurrence.** Nephrin tyrosine phosphorylation is induced and colocalizes with IgG (yellow) in the 1 h biopsy from the recurrent patient (patient 1). Induced nephrin tyrosine phosphorylation is not observed in the 1 h biopsy from the patient without recurrence (patient 2). Scale bar, 10 μm.

**Figure S3. Confocal images after dual staining for nephrin (red) and ShcA (green) from patients with and without recurrence.** ShcA is upregulated in the 1 h biopsy compared with the 0 h biopsy, and colocalizes with nephrin (yellow) in the recurrent patient (patient 1). Increased ShcA expression is not observed in the 1 h biopsy from the patient without recurrence (patient 2). Scale bar, 20 μm.

**Figure S4. Foot process width of patients with and without recurrence.** Average foot process width (FPW) was calculated as previously reported^4^. Average FPW of the recurrent patient increased in the 1 h biopsy specimen compared to the 0 h biopsy specimen. As reference, the mean value of average FPW of nine patients with primary FSGS in native kidney biopsy specimens is also shown (reproduced from our previous report^4^). Note that the FPW in the 1 h biopsy specimen of the recurrent patient was much smaller than those in the native kidney biopsies obtained from patients with primary FSGS, which reflected segmental, but not diffuse, foot process effacement in the 1 h biopsy specimen of the recurrent patient.

The error bar represents standard deviation.

**Figure S1**

**Patient 1 (recurrence)**

**
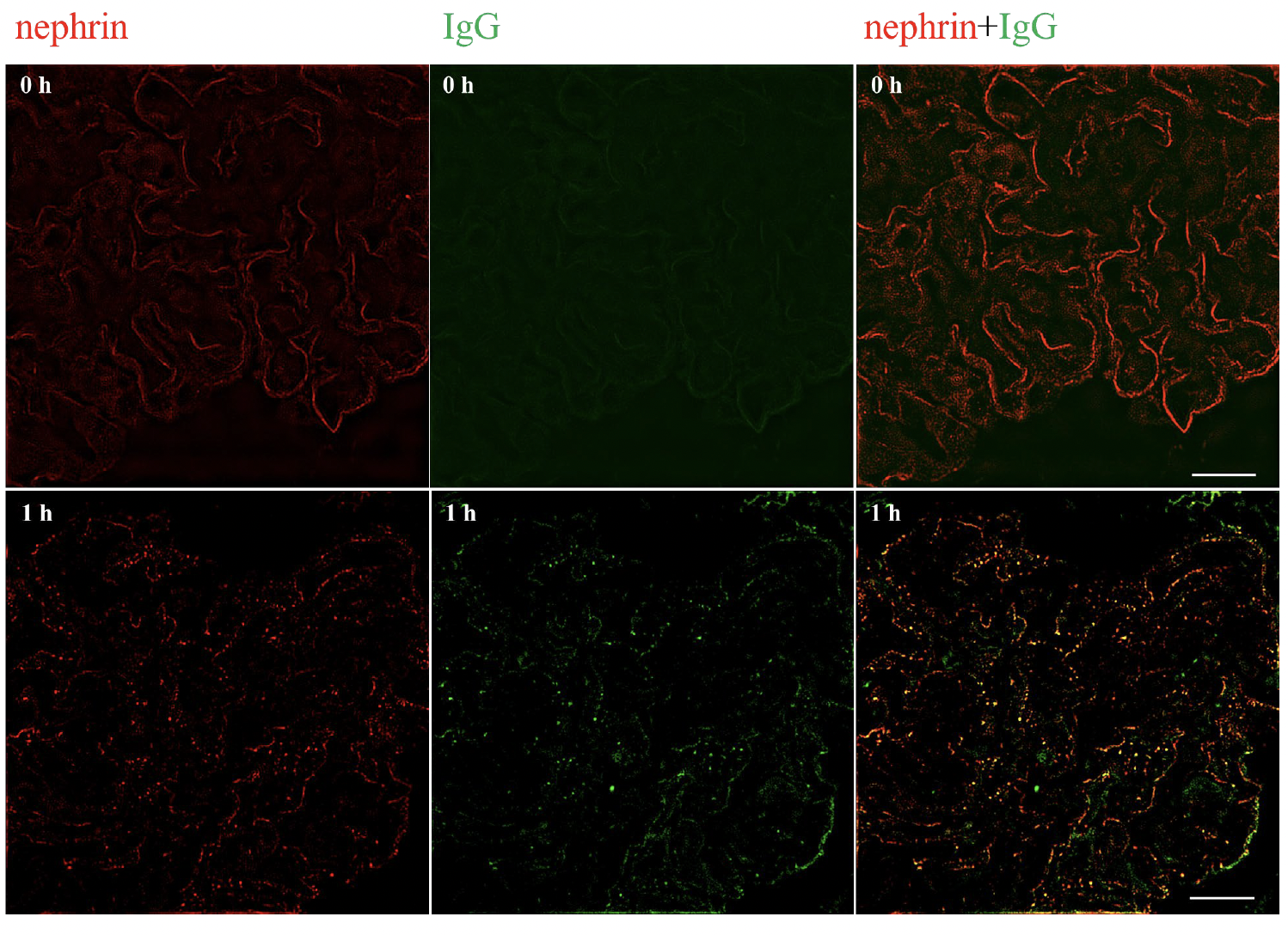
**

**Patient 2 (non-recurrence)**

**
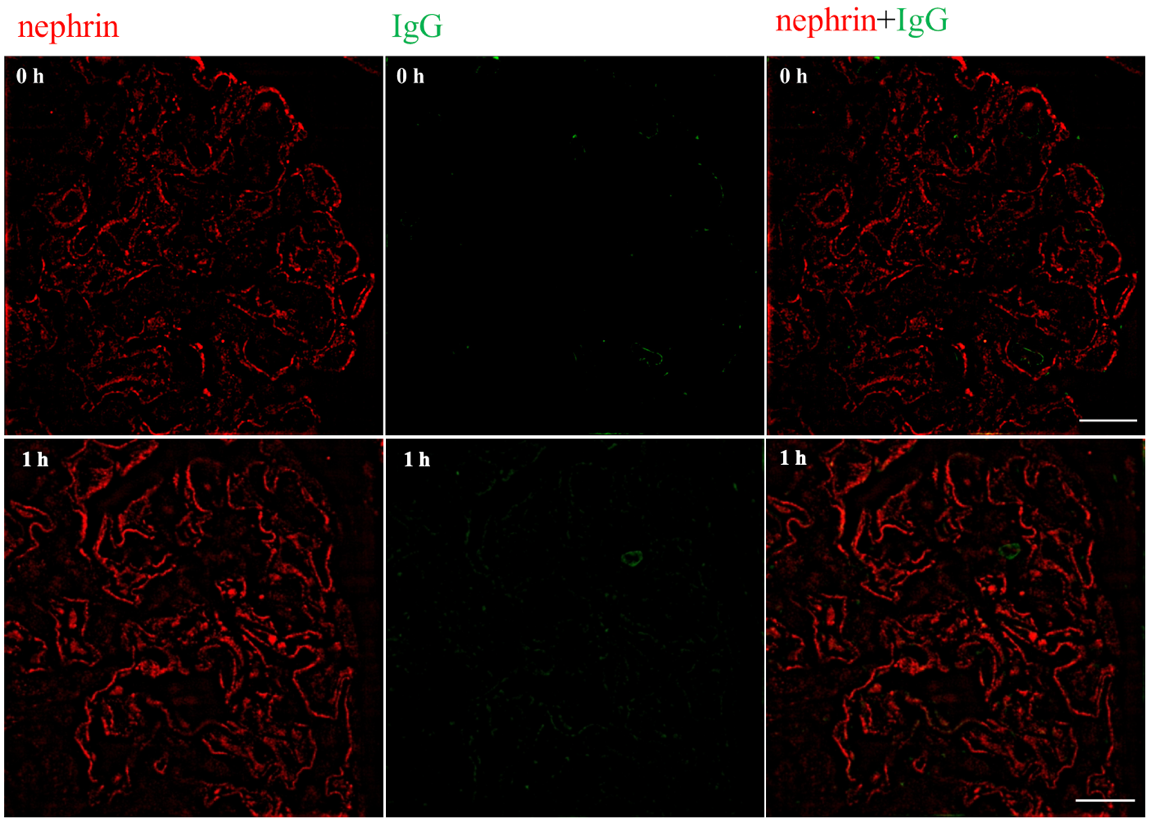
**

**Figure S2**

**Patient 1 (recurrence)**

**
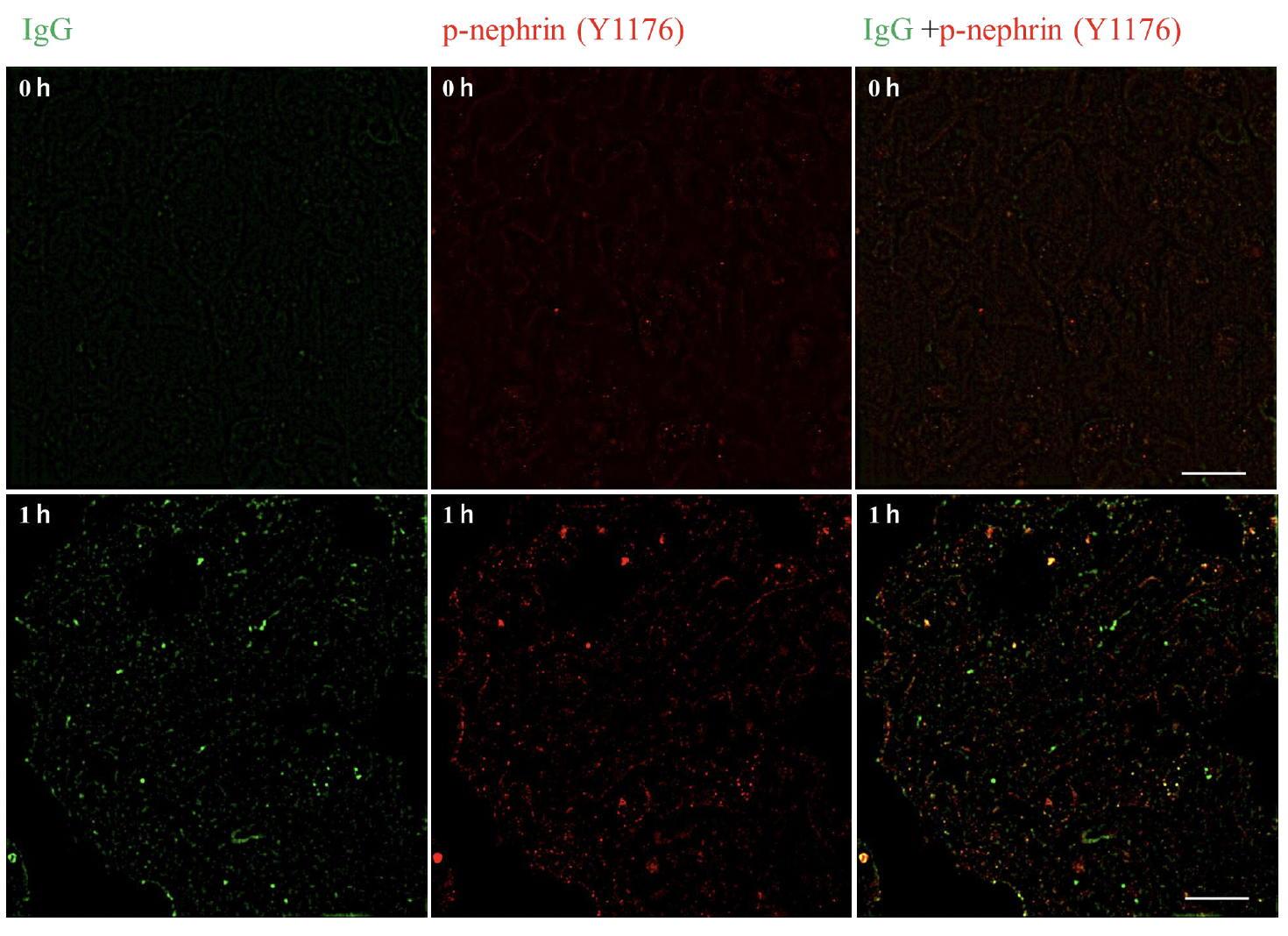
**

**Patient 2 (non-recurrence)**

**
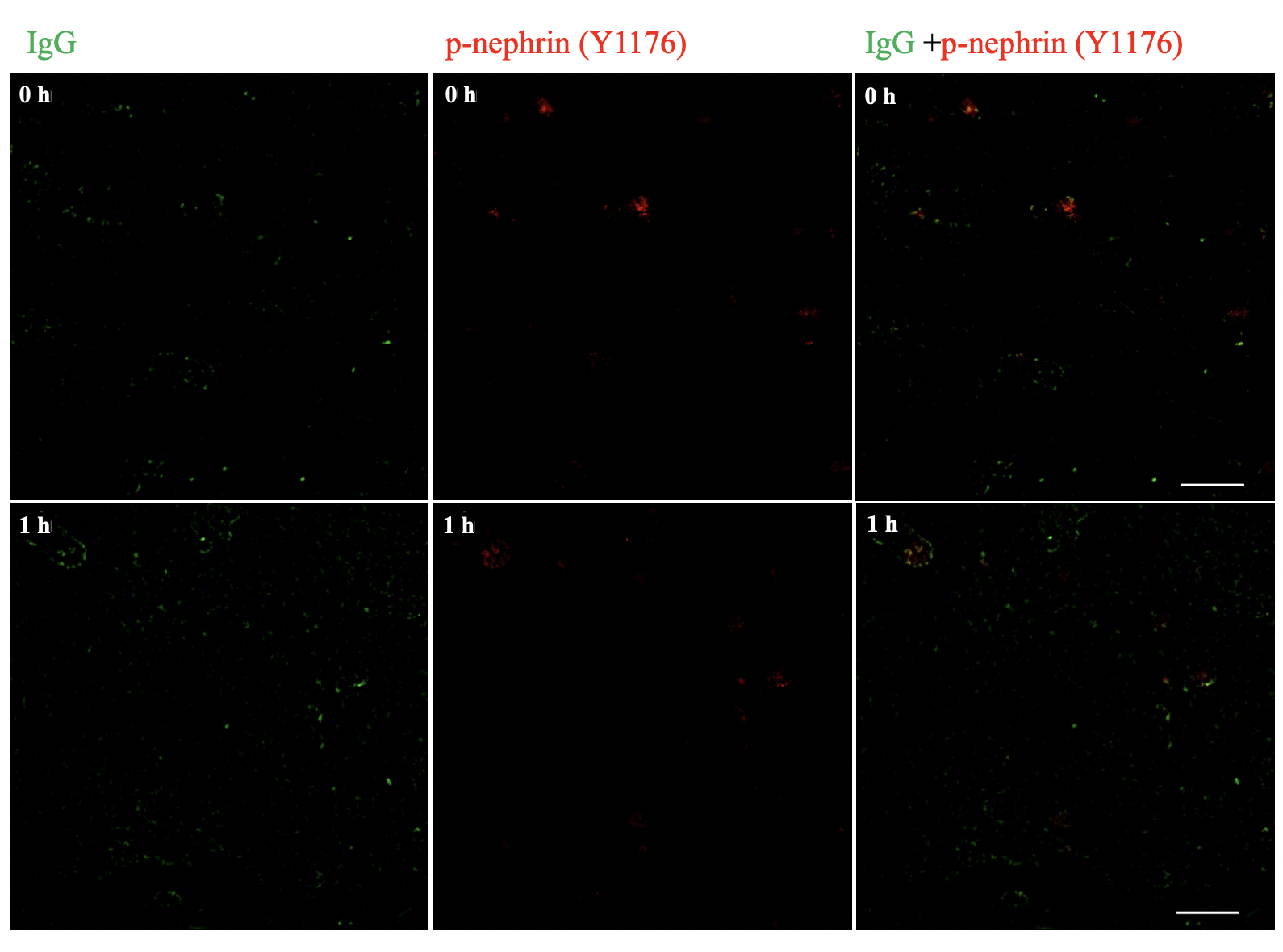
**

**Figure S3**

**Patient 1 (recurrence)**

**
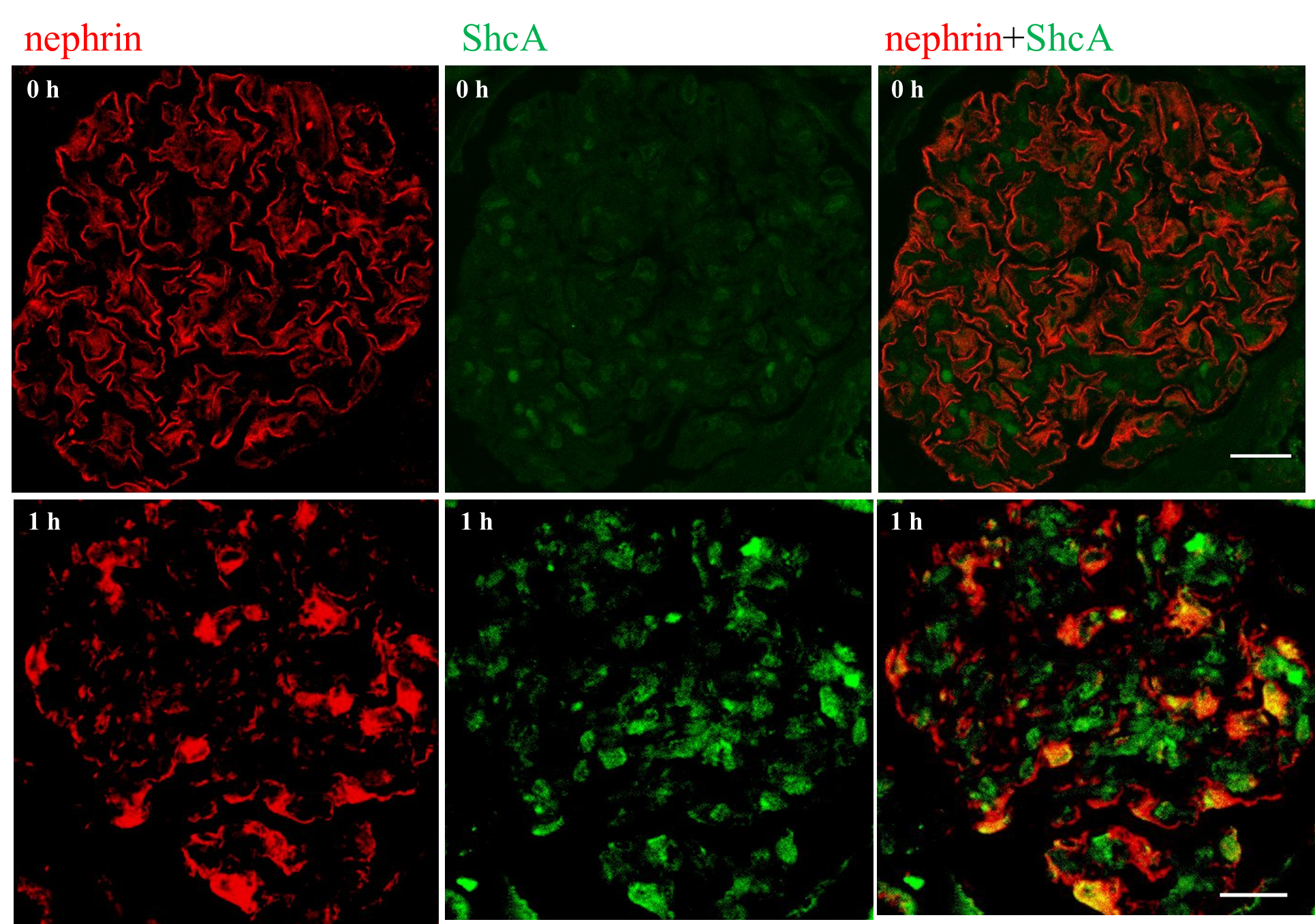
**

**Patient 2 (non-recurrence)**

**
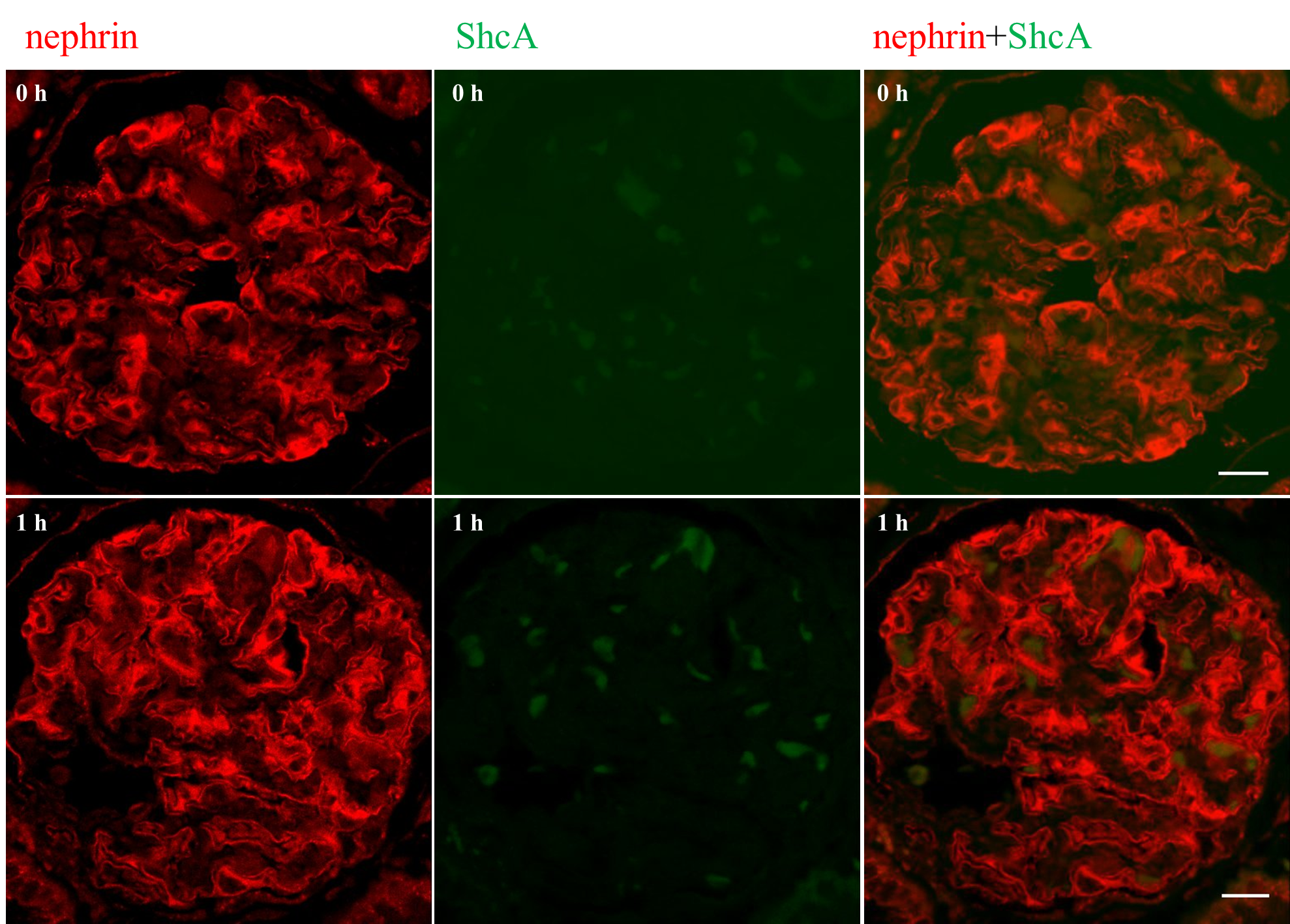
**

**Figure S4**

Average foot process width(nm)

**
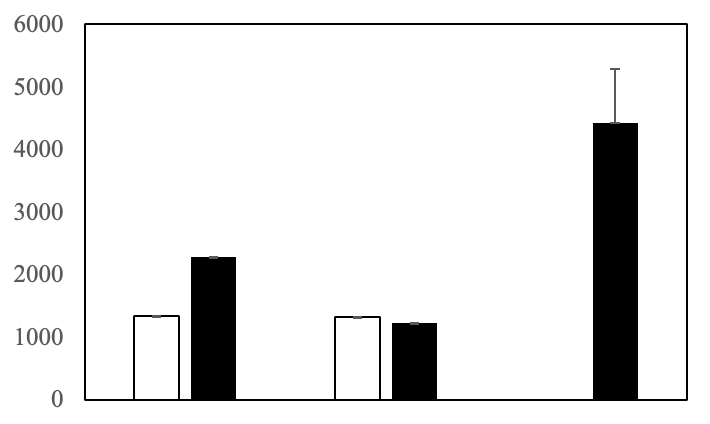
**

0h 1h 0h 1h primary FSGS

Patient 1 Patient 2 (native kidney)
